# Effects of lapatinib monotherapy: results of a randomised phase II study in therapy-naive patients with locally advanced squamous cell carcinoma of the head and neck

**DOI:** 10.1038/bjc.2011.237

**Published:** 2011-08-09

**Authors:** J M del Campo, R Hitt, P Sebastian, C Carracedo, D Lokanatha, J Bourhis, S Temam, D Cupissol, D De Raucourt, N Maroudias, C M Nutting, N Compton, D Midwinter, L Downie, N Biswas-Baldwin, I El-Hariry, K J Harrington

**Affiliations:** 1 Vall d’Hebron Hospital, Passeig Vall d’Hebron 119-12908035, Barcelona, Spain; 2 Hospital Universitario 12 de Octubre, Madrid 28041, Spain; 3 Medical College Regional Cancer Centre, Trivandrum, Kerala 695011, India; 4 Instituto de Enfermedades de Neoplásicas, Angamos Avenue East 2520, Lima 34, Peru; 5 Kidwai Memorial Institute of Oncology, Hosur Road, Bengaluru, Karnataka 560029, India; 6 Institut Gustave Roussy, 114 rue Edouard Vaillant 94805, Villejuif, France; 7 Le Centre Val d’Aurelle—Paul Lemarque, 31 Rue Croix Verte 34000, Montpellier, France; 8 Centre François Baclesse14000, Caen, France; 9 Appollonion Private Hospital, 20 Lefkotheou Avenue 2054, Strovolos, Nicosia, Cyprus; 10 The Royal Marsden Hospital, Fulham Road, London SW3 6JJ, UK; 11 GlaxoSmithKline, (I E-H formerly of GlaxoSmithKline) 980 Great West Road, Brentford, Middlesex TW8 9GS, UK

**Keywords:** epidermal growth factor receptor, lapatinib, squamous cell carcinoma

## Abstract

**Background:**

Lapatinib is a dual inhibitor of epidermal growth factor receptor (EGFR) and human EGFR-2 (HER-2) tyrosine kinases. This study investigated the pharmacodynamic and clinical effects of lapatinib in patients with locally advanced squamous cell carcinoma of the head and neck (SCCHN).

**Methods:**

In total, 107 therapy-naive patients with locally advanced SCCHN were randomised (2 : 1) to receive lapatinib or placebo for 2–6 weeks before chemoradiation therapy (CRT). Endpoints included apoptosis and proliferation rates, clinical response, and toxicity.

**Results:**

Versus placebo, lapatinib monotherapy did not significantly increase apoptosis detected by terminal deoxynucleotidyl transferase-mediated deoxyuridine triphosphate-biotin nick-end labelling or caspase-3 assays. A statistically significant decrease in proliferation using Ki67 assay was observed (*P*=0.030). In a subset of 40 patients that received ⩾4 weeks of lapatinib or placebo, objective response rate (ORR) was 17% (*n*=4/24) *vs* 0% (*n*=0/16). In the lapatinib single-agent responders, all had EGFR overexpression, 50% had EGFR amplification, and 50% had HER2 expression by immunohistochemistry (including one patient with HER2 amplification). However, these patients showed variable modulation of apoptosis, proliferation, and phosphorylated EGFR on drug treatment. Following CRT, there was a statistically non-significant difference in ORR between lapatinib (70%) and placebo (53%). There was no clear correlation between changes in apoptosis or proliferation and response to chemoradiation. Mucosal inflammation, asthenia, odynophagia, and dysphagia were the most commonly reported adverse events with lapatinib.

**Conclusion:**

Short-term lapatinib monotherapy did not demonstrate apoptotic changes, but provided evidence of clinical activity in locally advanced SCCHN, and warrants further investigation in this disease.

The epidermal growth factor receptor (EGFR) is involved in normal cell growth and differentiation. EGFR is particularly important in the pathogenesis of squamous cell carcinoma of the head and neck (SCCHN) (Kalyankrishna and Grandis, 2006), with reported overexpression in approximately 90% of tumours (Grandis and Tweardy, 1993). EGFR promotes growth and survival through several oncogenic signalling pathways, and its overexpression in SCCHN correlates with poor prognosis, short disease-free survival, and increased locoregional recurrence (Ang *et al*, 2002; Eriksen *et al*, 2004; Hitt *et al*, 2005; Chung *et al*, 2006), and makes it an attractive therapeutic target for therapy (Bonner *et al*, 2002; Johns *et al*, 2003; Harari *et al*, 2007).

Lapatinib is a reversible dual inhibitor of both EGFR and human EGFR-2 (HER2) tyrosine kinases, which in turn inhibits activation of downstream signalling pathways such as Erk1/2 and Akt in cell lines and xenografts (Xia *et al*, 2002; Rusnak *et al*, 2007). Lapatinib elicits cytostatic or cytotoxic anti-tumour effects, depending on the cell type (Chu *et al*, 2005; Coley *et al*, 2006; Rusnak *et al*, 2007; Konecny *et al*, 2008), and has demonstrated clinical activity in several solid tumours (Spector *et al*, 2005; Burstein *et al*, 2008; Cameron *et al*, 2008; Ravaud *et al*, 2008).

Lapatinib has shown a single-agent activity in *in vitro* and *in vivo* xenograft studies in human head and neck cancer cell lines (GSK, data on file). In addition, lapatinib has been safely combined with chemoradiation in phase I study, with a recommended dose of 1500 mg day^−1^ for future trials (Harrington *et al*, 2009). A randomised-phase II study of chemoradiation plus lapatinib, followed by maintenance lapatinib versus chemoradiation plus placebo, followed by placebo, has shown a statistically non-significant 17%-point superiority in favour of lapatinib-treated patients for complete and overall response at 6 months post-chemoradiation (Harrington *et al*, 2010). Lapatinib is currently in phase III trial with chemoradiation in patients with high-risk features after surgical treatment of stage III/IV head and neck cancer.

Although the molecular effects of many targeted-anticancer agents are often characterised *in vitro*, correlation of such effects with clinical outcome has only started to be adopted in proof-of-concept trials (Goulart *et al*, 2007; Banerji *et al*, 2008). Such an approach often uses apoptosis or proliferation endpoints (Sarker and Workman, 2007; Doroshow and Parchment, 2008). Spector *et al* (2005) have recently shown that the 3-week treatment with lapatinib in patients with advanced malignancies resulted in increased tumour cell apoptosis, occurring in patients with evidence of tumour regression. The objectives of this study were to explore the biological effects of lapatinib on apoptosis and proliferation in pre- and post-treatment tumour tissues in patients with locally advanced SCCHN.

## Materials and methods

This was a multinational, randomised, single-blinded, placebo-controlled study, conducted at 10 centres in six countries. The study was approved by independent ethics committees and regulatory agencies, and was carried out in accordance with the Declaration of Helsinki and good clinical practice. All patients gave written informed consent before enrolment.

Adults of at least 18 years of age with newly diagnosed stage III/IVA/IVB SCCHN undergoing chemoradiation therapy (CRT) were eligible. Other criteria included Eastern Cooperative Oncology Group performance status of 0, 1, or 2; adequate renal, hepatic, and bone marrow function; and normal left ventricular ejection fraction assessed by echocardiogram or multigated acquisition scan. Exclusion criteria included evidence of distant metastasis (stage IVC), earlier systemic chemotherapy, radiotherapy, or required concomitant use of cytochrome P450 3A4 inducers or inhibitors.

**Figure 1:**
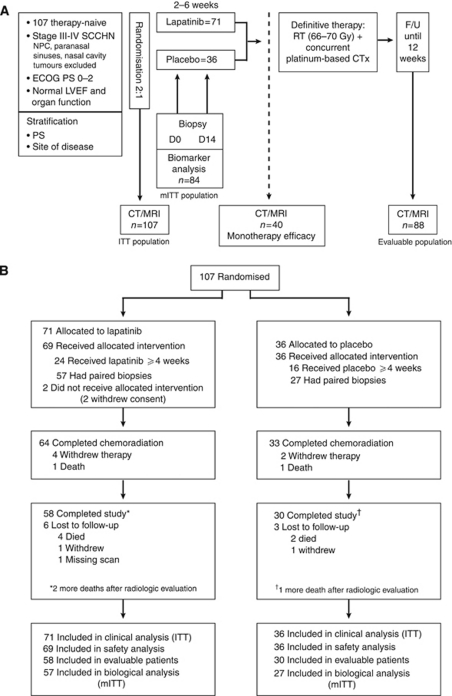
Study design and patient disposition. (**A**) Study design and allocated therapies are shown. (**B**) CONSORT diagram showing patient accountability. Abbreviations: CT=computed tomography; CTx=chemotherapy; ECOG=Eastern Cooperative Oncology Group; F/U=follow-up; ITT=intent-to-treat; LVEF=left ventricular ejection fraction; mITT=modified ITT; NPC=nasopharyngeal carcinoma; PS=performance status; RT=radiotherapy; SCCHN=squamous cell carcinoma of the head and neck.

Figure 1A shows the study design. Patients were randomised 2 : 1 to receive lapatinib (1500 mg d^−1^) or placebo, and stratified by tumour site and performance status. Treatment with lapatinib/placebo continued for 2–6 weeks until the start of CRT. The ‘window of opportunity’ represented the period of time required for radiotherapy preparation. The initiation of CRT was not delayed in patients receiving either lapatinib or placebo, as the monotherapy phase lasted no longer than local standards allowed. The mean time between commencing lapatinib/placebo and initiating CRT was 25.8 days for both arms. Chemotherapy schedule and CRT was mandated as concomitant cisplatin and radiation, and followed the local standard. Conventional radiotherapy was given to a total of 66–70 Gy given over 6–7 weeks.

Medications that inhibit or induce cytochrome P450 3A4 were prohibited. Lapatinib could be withheld for up to 1 week for any grade 3 or 4 toxicity, and permanently discontinued if grade 3 or 4 interstitial pneumonitis or cardiac dysfunction occurred.

### Study assessments

Baseline assessments included demography, medical history, physical examination, performance status, panendoscopy, echocardiogram, multigated acquisition scan, haematology, and clinical chemistry. Clinical examination and laboratory tests were repeated during treatment and at follow-up. Adverse events and serious adverse events were collected throughout the study and were graded using the National Cancer Institute's Common Terminology Criteria for Adverse Events, v 3.0 (Cancer Therapy Evaluation Program, 2006).

Objective response rate (ORR) was assessed by performing radiologic examination at baseline, 8 and 12 weeks post-CRT. Additional scans were performed before the commencement of CRT for patients receiving at least 4 weeks of lapatinib/placebo. Efficacy was defined according to Response Evaluation Criteria in Solid Tumours criteria, version 1.0. (Therasse *et al*, 2000) All scans and clinical data were reviewed centrally by an independent review board (BioClinica, Newtown, PA, USA), and all readers were blinded to treatment. Follow-up beyond 3 months post-CRT was not part of the protocol.

A positron emission tomography (PET) substudy was conducted at participating centres. Thirty-five subjects who participated in the trial agreed to the substudy with fluorodeoxyglucose (FDG)–PET imaging at baseline and the follow-up time point. The sites that were qualified to conduct FDG–PET scans adhered to guidelines regarding the scans. The first scan of the head and neck was performed at screening, before biopsy, to obviate effects of the biopsy procedure on glucose uptake. The second scan was conducted at the end of the week-2 lapatinib treatment (again, before biopsy). The procedure further included: (1) administration of approximately 300–370 MBq FDG; (2) 60 minutes ±10 minutes of rest; (3) an attenuation scan for transmission correction; (4) whole-body emission scan; and (5) whole-body postcontrast computed tomography scan.

Analysis included acquisition of both quantitative measurements of standard uptake values (SUVs), as well as a qualitative assessment. The target and non-target lesions were analysed, based on SUV levels by a PET nuclear medicine expert. At baseline and post-monotherapy time points, approximately 300–370 MBq FDG was administered. Acquisition of SUV occurred after 60 minutes (±10 minutes) of rest.

### Tissue acquisition

Fresh tumour biopsies were obtained at time of study enrolment (day 0) and after 2 weeks of study participation (day 14). Biopsies were immediately fixed in 10% neutral-buffered formalin containing phosphatase inhibitors before paraffin-embedded sections were prepared. Hematoxylin and eosin staining was used to confirm the presence of tumour.

### Biological evaluations

Apoptosis was measured using immunohistochemistry (IHC) in paired tumour samples by both terminal deoxynucleotidyl transferase-mediated deoxyuridine triphosphate nick-end labelling (TUNEL) and active caspase-3 assays. The expression patterns of Ki-67, EGFR, HER2, phosphorylated-EGFR (pEGFR), p16, and p53 were also studied by IHC (details of antibody tests can be found in Supplementary Table 1). HER2 and EGFR gene amplification were assessed using fluorescence *in situ* hybridisation.

### Statistical analysis

The primary endpoint was apoptotic index (AI), which was calculated at baseline and after treatment (percent stained nuclei/total nuclei). Secondary endpoints included proliferation rate given by proliferation index (PI) (percent proliferating cells/total number of cells), ORR, adverse events, and correlative biomarker analyses.

Using a two-sided significance level of 5 and 80% power, 90 patients were required to detect a 24% difference in apoptosis between the two groups. To allow for dropouts during tissue acquisition, a total of 107 patients were randomised.

The change from baseline in AI was analysed using an analysis of covariance model, adjusted for the baseline strata. All other endpoints were summarised as appropriate.

All clinical and biological analyses presented were based on the intent-to-treat (ITT) population, which comprised all patients randomised to study treatment. The evaluable population for radiologic assessment was defined as all patients who completed CRT and had baseline and follow-up scans. This population was used for a more accurate estimation of clinical benefit.

## Results

### Patient characteristics

Between March 2006 and July 2007, a total of 107 patients were randomised to receive lapatinib (*n*=71) or placebo (*n*=36). The study reached the last patient's last visit in December 2007. Figure 1B shows the flow of patients through the trial and highlights the specific patient populations that will be discussed throughout the manuscript: the ITT population comprised 107 patients randomised to either lapatinib or placebo; the modified ITT population included 84 patients in whom pre- and post-treatment biopsies were obtained for analysis of biological endpoints; the evaluable population was made up of 88 patients who had CT/MRI scans at 12 weeks post-chemoradiation, and who were evaluable for treatment response; the monotherapy efficacy population comprised a subgroup of 40 patients who underwent CT/MRI scanning after receiving at least 4 weeks of lapatinib/placebo before chemoradiation. A total of 84 and 88 patients were considered evaluable for apoptosis/proliferation and post-CRT clinical activity, respectively. Table 1 shows the baseline demography. The two treatment groups were generally well balanced. The incidence of p16-positive tumours (IHC 2+/3+) was 43% (35% for the lapatinib group and 60% for the placebo group). EGFR overexpression (IHC 2+/3+) was seen in 93 and 83% of lapatinib and placebo patients, respectively; EGFR gene amplification was seen in 28 and 39% of patients. EGFR amplification was highest in hypopharyngeal tumours (*n*=8/13 (62%)), and lowest in oral cavity (16%) and laryngeal tumours (17%). HER2 overexpression and gene amplification accounted for only 7% and 4% in total, respectively.

Eighty-two percent of lapatinib and 92% of placebo patients were at least 80% compliant. The planned radiotherapy dose was given to 88 and 81% of lapatinib and placebo patients, respectively. For the lapatinib and placebo groups, the median radiation doses delivered were 70.0 and 68.0 Gy, respectively. Similarly, 93 and 92% of patients in the lapatinib and placebo groups, respectively, completed at least two cycles of planned chemotherapy. The median cisplatin doses for lapatinib and placebo groups were 248.6 and 242.6 mg m^−2^, respectively. In regard to compliance with study drugs, the mean cumulative dose for lapatinib was 35 217.4 mg with a median value of 30 000.0 mg. The corresponding mean and median values for placebo were 34 916.7 and 31 500.0 mg, respectively.

### Biological effects of lapatinib

The activation of EGFR (pEGFR, IHC 2+/3+) was observed in 28 (41%) and 15 (42%) patients in the lapatinib and placebo arms (Table 1), of which 24 and 15 patients, respectively, had post-treatment data available. Lapatinib reduced the level of pEGFR in 63% (*n*=15/24) of cases, versus 33% (*n*=5/15) in the placebo group (*P*=0.11).

#### Apoptosis induction

**Figure 2:**
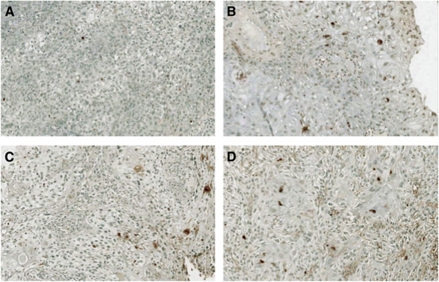
AI by TUNEL assay. Representative IHC TUNEL staining pretreatment (**A**) and posttreatment (**B**) with lapatinib, and pretreatment (**C**) and posttreatment (**D**) with placebo.

Apoptotic cells detected by TUNEL were frequent with lapatinib and placebo, both pre- and posttreatment (Figure 2A–D). Both groups showed similar rates of pretreatment apoptosis, with an AI mean of 3.8 (s.d.=3.38) and 3.2% (s.d.=3.14) for lapatinib and placebo, respectively. The post-treatment mean AI was 8.0% (s.d.=6.66) with lapatinib and 9.4% (s.d.=12.22) with placebo. The change in AI from pretreatment was not statistically significant for lapatinib versus placebo at the two-sided 5% significance level (difference=−1.7, s.e.=1.93, *P*=0.394, 95% confidence interval: −5.50%, 2.19% see Supplementary Figure 1). AI was compared with T-stage, stage of disease, and tumour site; the results are shown in Supplementary Figures 2–4. In the caspase-3 assay, the change from pretreatment values with lapatinib was not statistically significant. The values for mean change in caspase-3 from baseline to post-treatment were 3.0 (s.d.=3.4) and 1.6 (s.d.=3.8) for the lapatinib- and placebo-treated patients, respectively. Data for AI from caspase-3 and TUNEL assays were concordant (data not shown).

#### Effect of lapatinib on proliferation

**Figure 3:**
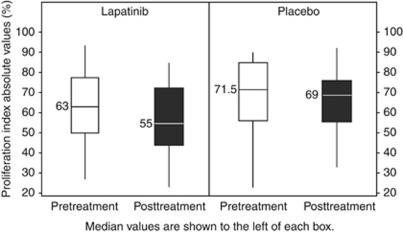
Effect of lapatinib on proliferation determined by Ki-67; box whisker plot of pretreatment and posttreatment proliferative index with lapatinib and placebo. The median values are presented to the left of each box. The mean values were: lapatinib pretreatment −62.7%, posttreatment −56.7% placebo pretreatment −66.1%, posttreatment −64.4%.

The pretreatment PI mean was 62.7 (s.d.=17.54) and 66.1% (s.d.=19.47) with lapatinib and placebo, respectively. Treatment with lapatinib decreased the PI, compared with placebo, with a mean of 56.7 (s.d.=17.49) and 64.4% (s.d.=16.54), respectively. The relative change from pretreatment was statistically significant at the two-sided 5% significance level (difference: −5.4%, s.e.=2.47, *P*=0.030, 95% confidence interval: −10.36% to −0.53% Figure 3).

### Clinical outcome

**Figure 4:**
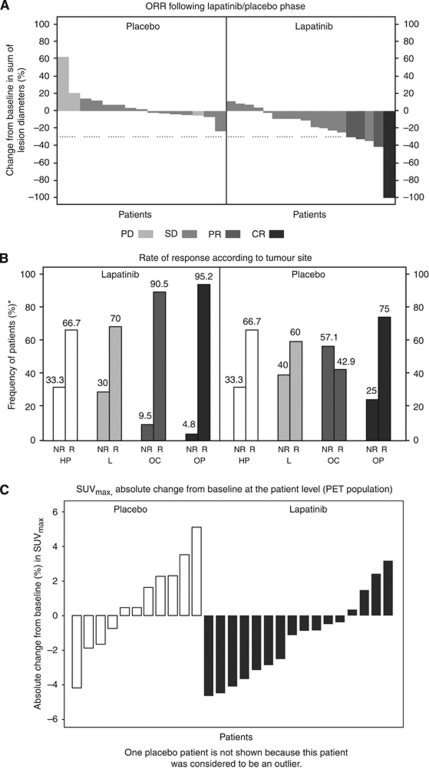
(**A**) Waterfall plot of independently assessed ORR following lapatinib/placebo phase; (**B**) Bar chart of ORR following CRT versus tumour site; (**C**) Waterfall plot of the change from baseline in independently read SUV_max_ measurements. Abbreviations: R=responders (complete and partial response); NR=nonresponders (stable and progressive disease); HP=hypopharynx; L=larynx; OC=oral cavity; OP=oropharynx. ^*^Percent within levels of tumour site.

Forty patients were assessed for response after the monotherapy phase and before CRT. The independently assessed ORR was 17 (*n*=4/24) versus 0% (*n*=0/16) in the ITT and evaluable populations, respectively, following approximately 4 weeks of lapatinib/placebo treatment. No progressive disease was observed with lapatinib, compared with 25% with placebo (see Table 2 and Figure 4A). From the lapatinib responders, all patients had EGFR overexpression; 50% had EGFR amplification; 50% HER2 expression by IHC, in one of these cases, HER2 gene was amplified; and variable modulation of apoptosis, proliferation, and pEGFR (details in Supplementary Table 2). Two of the responders were positive for p16.

The independently assessed ORR following CRT in the evaluable population was 86 and 63% in the lapatinib and placebo arms, respectively (see Table 2). Moreover, in the ITT, the response rate was 70 and 53%. A higher number of patients presented with progressive disease in the placebo arm (25%) than in the lapatinib arm (6%). Oropharyngeal and oral cavity tumours were characterised by highest response rate (95% and 91%, respectively) in the lapatinib arm, compared with larynx (70%), hypopharyngeal tumours (67%), or placebo arm (67%, 43%, 60%, and 67%, respectively; see Figure 4B).

In the PET substudy (*n*=35), maximum SUV (SUV_max_) was reduced from baseline values in 75% of patients treated with lapatinib compared with 36% treated with placebo (see Figure 4C). The change in median SUV_max_ was −12.9 for lapatinib and +6.1 for placebo. The mean SUV change from baseline was also assessed, with a median −10.9 for lapatinib and +3.2 for placebo.

### Safety

In the monotherapy phase, only patients receiving lapatinib reported rash (23%) and diarrhoea (22%), which were mainly grades 1 and 2 (see Supplementary Figure 5). Throughout the complete duration of the study, the lapatinib/CRT group showed a higher incidence of diarrhoea (26%) compared with 6% in the placebo/CRT group, and a slightly higher incidence of mucosal inflammation (70%) compared with 67% in the placebo/CRT group (see Supplementary Table 3). Grade 3 and 4 mucositis (a grouping of associated adverse events) were also higher in the lapatinib/CRT arm (46% and 4%, respectively), compared with placebo (41% grade 3; no grade 4 events).

The reported serious adverse events during or after CRT were higher in the placebo/CRT group (36%) than in the lapatinib/CRT group (19%), with mucosal inflammation, the most common serious adverse event (6% and 4%, respectively; see Supplementary Table 4).

Seven patients experienced cardiac-related events: 2 (3%) in the lapatinib arm and 5 (14%) in the placebo arm. The event was fatal for two patients (*n*=1 in each arm).

Twelve deaths were reported (10 and 14% of patients in the lapatinib and placebo arms, respectively). The primary causes were disease under study (6% lapatinib and 8% placebo), ventricular fibrillation (*n*=1, placebo), septicaemia (3%, placebo), and intestinal perforation, cardiac arrest, and sudden death (3% each, lapatinib). None of these fatal events was attributed to the study medication.

## Discussion

This study set out to investigate whether the biological effects of lapatinib on cell survival and growth pathways predict clinical outcome and to identify any subgroup of patients that may benefit from lapatinib treatment. It was previously shown that activated EGFR modulates the proapoptotic and antiapoptotic pathways (Modjtahedi *et al*, 1998; Grandis *et al*, 2000; Ginsberg, 2007; Goel *et al*, 2007). Therefore, the primary objective assessed the effect of lapatinib on apoptosis. Apoptosis induction was not statistically different compared with placebo, and therefore, the primary endpoint was not met. Indeed, the AI by TUNEL staining increased, following both lapatinib and placebo treatment. Although the possibility that these data may have been affected by artifact introduced during post-treatment biopsy handling should be considered; we believe that the randomised nature of the study should have provided insurance against this risk. The lack of a clear apoptotic signal may be somewhat surprising, as lapatinib is a potent inhibitor of pEGFR and pHER2 in cell-free systems (Rusnak *et al*, 2001), and induces apoptosis in *in vitro* and *in vivo* models (Xia *et al*, 2002; Zhou *et al*, 2006) as well as clinical studies (Spector *et al*, 2005). However, similar values have previously been reported for spontaneous apoptosis and apoptosis induction (Hotz *et al*, 1999; Grabenbauer *et al*, 2003). Unfortunately, results regarding the prognostic and predictive significance of apoptosis are rather conflicting in SCCHN (Bartelink *et al*, 1999; Tsuchiya *et al*, 2001; Grabenbauer *et al*, 2003), which could be attributed, in part, to the methodologic complexities associated with these assays and the inherently asynchronous apoptotic process within a given tissue (Potten, 1996). TUNEL detects late apoptotic events, and despite the fact that positive signals are also found in necrotic cells and in some cells in which DNA fragmentation is later reparable (Collins *et al*, 1997), the co-analysis of the nuclear morphology provides a relatively accurate indicator of apoptosis (Sheridan *et al*, 1999; Willingham, 1999). Caspase-3 activation is an early event in both the intrinsic and extrinsic apoptotic pathways (Kaufmann and Vaux, 2003); nevertheless, the assay may not detect apoptosis independent of mitochondrial pathways (Gown and Willingham, 2002). The lack of apoptotic changes despite the observation of tumour shrinkage in some patients suggests that caspases may not be involved in lapatinib-induced cell death or that lapatinib does not induce apoptosis, and its mechanism of action is through some other pathway in locally advanced SCCHN.

Other biological effects with lapatinib treatment are intriguing and warrant further investigation. For example, the inhibition of pEGFR activity within 2 weeks of lapatinib treatment and the significant decrease in the proliferative activity may, in part, have contributed to the higher response rates to subsequent CRT documented in lapatinib-treated patients. This observation also supports a possible role for lapatinib in combination with radiotherapy or CRT as a means of preventing accelerated repopulation (Harrington *et al*, 2007). However, these data must be interpreted in light of the fact that 33% of placebo-treated patients also showed evidence of reduction of the pEGFR level. Future studies should address the issue of the reproducibility of these assays on serial samples.

Despite ambiguous biological results, this study conducted in a treatment-naive population with locally advanced disease showed an ORR of 17% after the monotherapy phase, including one complete response. Furthermore, there was a larger proportion of lapatinib-treated patients with a reduction in SUV_max_ in the PET substudy. Given the short lapatinib treatment, these findings are promising and indicate lapatinib activity in locally advanced SCCHN. These findings oppose a recent study by Abidoye *et al*, (2006) that reported minimal clinical activity in patients treated with lapatinib, who had recurrent or metastatic SCCHN.

Interestingly, all four monotherapy responders had tumours in which EGFR was overexpressed, two of which also showed HER2 expression and one that showed HER2 gene amplification. None of the other biological characteristics were predictive of response to lapatinib. However, the number of responding patients is too small to make any meaningful conclusions. More importantly, the post-treatment biopsy was obtained at earlier time points (day 14±3 days) than the radiologic scans (week 4 approximately), which may provide an explanation of why apoptotic changes were not also observed.

The ORR following CRT was higher in the lapatinib group compared with placebo in the ITT and evaluable populations. The fact that there was an excess of HPV-positive patients in favour of the placebo-treated group means that the absolute difference may have been greater if the study arms had been perfectly balanced. Although this study was not powered to test for differences in ORR between the study arms, the data suggest that lapatinib is worthy of further evaluation in patients with locally advanced head and neck cancer. The short duration of lapatinib before commencing CRT may have provided insufficient suppression of EGFR signalling to induce apoptosis, but may prime tumour cells for subsequent CRT-induced cell death through an unknown mechanism. This effect is unlikely to have been due to drug-induced G1 cell cycle arrest. Given the responses observed following monotherapy, the reduced tumour volume before commencing CRT in the lapatinib group may also provide an explanation.

Although EGFR overexpression seems to be predictive of response to lapatinib or lapatinib/CRT, EGFR gene amplification was not, which is in contrast to various reports with other EGFR inhibitors in SCCHN (Cohen *et al*, 2005; Erjala *et al*, 2006; Agulnik *et al*, 2007; Thomas *et al*, 2007). Although no clear correlation was demonstrated between HER2 overexpression and response to lapatinib or lapatinib/CRT, a functional role through heterodimerisation with EGFR cannot be excluded. Of interest is that two of the four monotherapy responders showed both EGFR and HER2 coexpression. The patient with 3+ overexpression for both EGFR and HER2 also demonstrated gene amplification for both receptors. Similar to a previous study (Harrington *et al*, 2009), the treatment was well tolerated and did not lead to significant modifications of CRT. The majority of patients received the planned radiotherapy and chemotherapy, which was generally similar to that seen in other studies (Cooper *et al*, 2004). Furthermore, an independent data-monitoring committee, which evaluates the safety in several ongoing phase II and III trials of lapatinib in SCCHN, has raised no safety concerns in this regard.

The present study provides a useful design that allowed inter- and intrapatient evaluation of the pharmacodynamic effects and putative predictors of response to targeted agents. It showed the feasibility of obtaining paired biopsies in locally advanced SCCHN. However, some limitations of study design should be addressed. First, there is no consensus on the appropriate timing for the second biopsy; therefore, the timing for this study was empirically chosen. Hence, the observed effects may not reflect the actual molecular events that lead to apoptosis or growth arrest. Second, the small number of patients means the results should be interpreted with caution. Third, there is a fairly short follow-up period, which did not allow for assessment of survival.

In summary, a short treatment period with lapatinib suggests that it may 1) not affect apoptosis; 2) lead to inhibition of pEGFR; 3) decrease proliferation; 4) induce tumour regression and enhance response to CRT; and 5) cause oropharyngeal and oral cavity tumours to respond favourably to treatment. It would be of value to confirm these hypotheses with large cohorts of patients. Work is ongoing to investigate other EGFR family members and their effect on downstream signalling pathways.

## Figures and Tables

**Table 1:** Baseline patient characteristics (ITT population)

**Characteristic**	**Lapatinib (*n*=71)**	**Placebo (*n*=36)**
*Age, years*		
Median (range)	58 (33–80)	55 (37–78)
		
*Sex,* n (%)		
Female	16 (23)	4 (11)
Male	55 (77)	32 (89)
		
*ECOG performance status,* n (%)		
0–1	70 (99)	35 (97)
2	1 (1)	1 (3)
		
*Primary tumour site,* n (%)		
Oral cavity	27 (38)	7 (19)
Oropharynx	24 (34)	16 (44)
Larynx	12 (17)	6 (17)
Hypopharynx	8 (11)	7 (19)
		
*Histologic grade at initial diagnosis,* n (%)		
Well differentiated	24 (34)	11 (31)
Moderately differentiated	21 (30)	11 (31)
Poorly differentiated	8 (11)	4 (11)
Can not be assessed	17 (24)	9 (25)
Missing	1 (1)	1 (3)
		
*T-category,* n (%)		
T1	1 (1)	0
T2	10 (14)	6 (17)
T3	26 (37)	10 (28)
T4	34 (48)	20 (56)
		
*N-category,* n (%)		
N0	19 (27)	3 (8)
N1	12 (17)	8 (22)
N2	39 (55)	23 (64)
N3	1 (1)	2 (6)
		
*TNM staging*, n (%)		
III	20 (28)	7 (19)
IV	51 (72)	29 (81)
		
*p16 expression by IHC,* n (%)		
*n*	66	30
0, 1+	34 (52)	10 (33)
2+, 3+	23 (35)	18 (60)
Missing	9 (14)	2 (7)
		
*EGFR protein expression by IHC,* n (%)		
*n*	69	36
0, 1+	4 (6)	4 (11)
2+, 3+	64 (93)	30 (83)
Missing	1 (1)	2 (6)
		
*pEGFR expression by IHC,* n (%)		
*n*	69	36
0, 1+	40 (58)	19 (53)
2+, 3+	28 (41)	15 (42)
Missing	1 (1)	2 (6)
		
*EGFR gene amplification by FISH,* n (%)		
*n*	67	33
Amplified	19 (28)	13 (39)
Oral cavity	3/25 (12)	2/7 (29)
Oropharynx	9/23 (39)	7/15 (47)
Larynx	2/11 (18)	1/6 (17)
Hypopharynx	5/8 (63)	3/5 (60)
Not amplified	48 (72)	20 (61)
Oral cavity	22/25 (88)	5/7 (71)
Oropharynx	14/23 (61)	8/15 (53)
Larynx	9/11 (82)	5/6 (83)
Hypopharynx	3/8 (38)	2/5 (40)
		
*HER2 expression by IHC,* n (%)		
*n*	69	36
0, 1+	64 (93)	31 (86)
2+, 3+	4 (6)	3 (8)
Missing	1 (1)	2 (6)
		
*HER2 gene amplification by FISH,* n (%)		
*n*	65	33
Amplified	2 (3)	2 (6)
Not amplified	63 (97)	31 (94)
		
*p53 expression by IHC,* n (%)		
*n*	69	36
0, 1+	39 (57)	19 (53)
2+, 3+	30 (43)	17 (47)

Abbreviations: ECOG=Eastern Cooperative Oncology Group; EGFR=epidermal growth factor receptor; FISH=fluorescent *in situ* hybridisation; HER2=human EGFR receptor-2; IHC=immunohistochemistry; pEGFR=phosphorylated EGFR; TNM=tumour, node, metastasis.

**Table 2:** Objective response rate

	**Investigator evaluation (ITT)**		**Independent evaluation (ITT)**		**Evaluable population^a^**	
	**Lapatinib *n*=71**	**Placebo *n*=36**	**Lapatinib *n*=71**	**Placebo *n*=36**	**Lapatinib *n*=71**	**Placebo *n*=36**
*Response rate following lapatinib/placebo phase,* n (%)						
*n*	24	16	24	16	19	16
CR	1 (4)	0	1 (4)	0	1 (5)	0
PR	5 (21)	1 (6)	3 (13)	0	3 (16)	0
ORR	6 (25)	1 (6)	4 (17)	0	4 (21)	0
SD	11 (46)	12 (75)	15 (63)	12 (75)	15 (79)	12 (75)
PD	2 (8)	3 (19)	0	4 (25)	0	4 (25)
Non-evaluable^b^	5 (21)	0	5 (21)	0	—	—
						
*Response rate following CRT,* n (%)						
*n*	71	36	71	36	58	30
CR	29 (41)	10 (28)	16 (23)	2 (6)	16 (28)	2 (7)
PR	21 (30)	9 (25)	34 (48)	17 (47)	34 (59)	17 (57)
ORR	50 (70)	19 (53)	50 (70)	19 (53)	50 (86)	19 (63)
SD	6 (8)	4 (11)	4 (6)	2 (6)	4 (7)	2 (7)
PD	2 (3)	7 (19)	4 (6)	9 (25)	4 (7)	9 (30)
Withdrawn	7 (10)^c^	1 (6)^d^	7 (10)^c^	1 (6)^d^	—	—
Non-evaluable	6 (8)^e^	5 (14)	6 (8)^e^	5 (14)^f^	—	—
						
**Correlation of independent ORR post-CRT with EGFR expression in evaluable patients, *n* (%)**						
	**EGFR (IHC)**		**pEGFR at screening**		**EGFR (FISH)**	
*Low expression (0, 1+)/ non-amplified*	*n*=2	*n*=4	*n*=34	*n*=16	*n*=42	*n*=17
ORR	1 (50)	3 (75)	29 (85)	10 (63)	37 (88)	11 (65)
SD	0	0	3 (9)	1 (6)	4 (10)	1 (6)
PD	1 (50)	1 (25)	2 (6)	5 (31)	1 (2)	5 (29)
						
*Overexpression (2+, 3+)/ amplified*	*n*=55	*n*=25	*n*=23	*n*=13	*n*=15	*n*=11
ORR	48 (87)	15 (60)	20 (87)	8 (62)	12 (80)	7 (64)
SD	4 (7)	2 (8)	1 (4)	1 (8)	0	1 (9)
PD	3 (5)	8 (32)	2 (9)	4 (31)	3 (20)	3 (27)

Abbreviations: CR=complete response; CRT=chemoradiation therapy; EGFR=epidermal growth factor receptor; FISH=fluorescent *in situ* hybridisation; IHC, immunohistochemistry; PD=progressive disease; ITT=intent-to-treat; ORR=objective response rate; pEGFR=phosphorylated EGFR; PR=partial response; SD=stable disease.

a Patients completed CRT and have radiologic scans at baseline and follow-up.

b CRT started before assessment: two PR after 8 Gy and 16 Gy, and three SD after 8–22 Gy.

c Five patients died after CRT, one patient withdrew after CRT, and one had an unreadable scan.

d One patient completed CRT, but had no available scans.

e Four patients withdrew before completing CRT, two patients never started CRT.

f One patient withdrew before completing CRT, one patient died, and three patients did not have scans.
