# Supplementary figures and images for: Effects of lapatinib monotherapy: results of a randomised phase II study in therapy-naive patients with locally advanced squamous cell carcinoma of the head and neck
Source: Br J Cancer. 2011 Aug 9;105(5):618–27. doi: 10.1038/bjc.2011.237 (PMC3188940; doi:10.1038/bjc.2011.237)

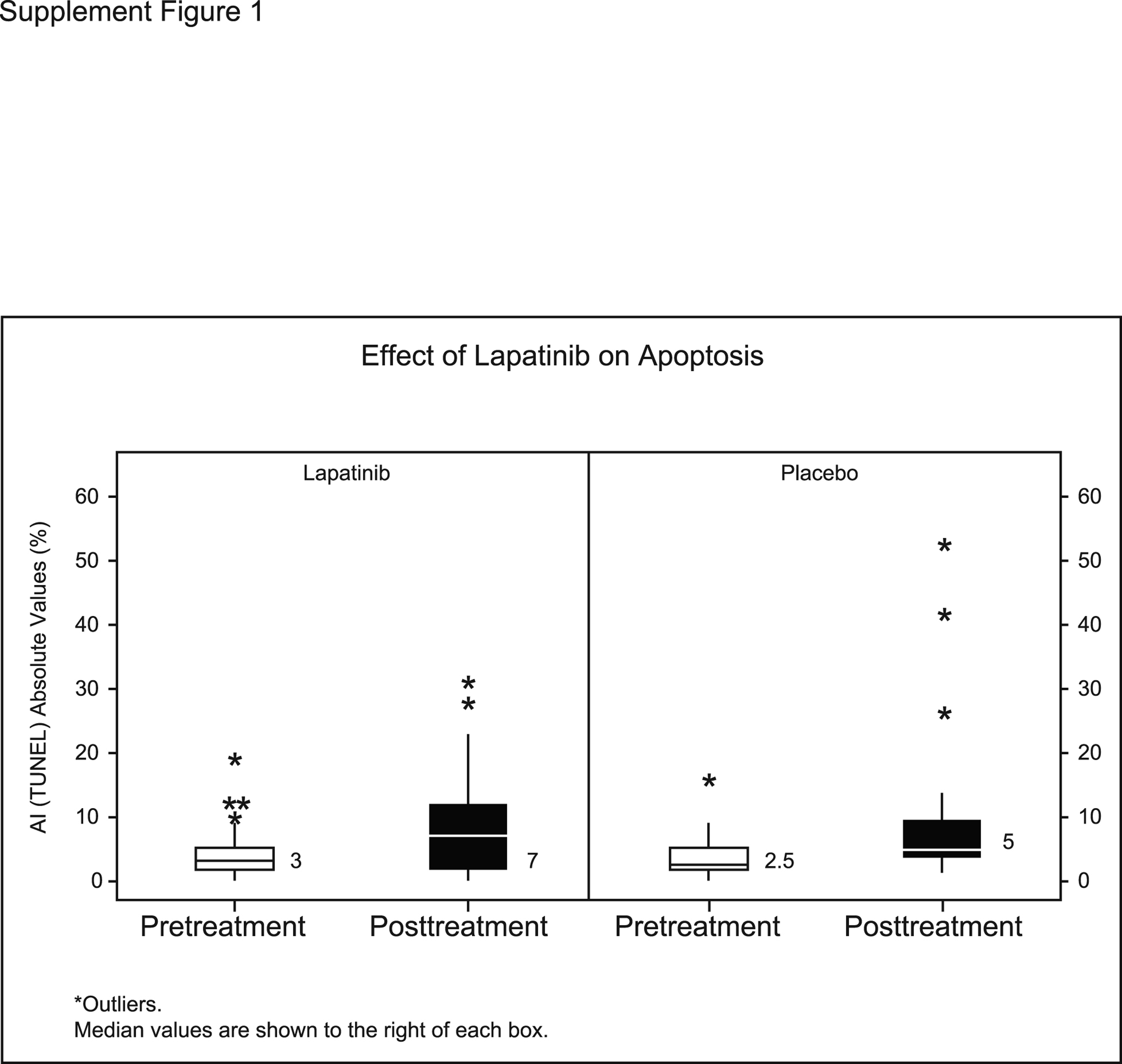

Supplement: Supplementary Figure 1 [file bjc2011237x1.tif]

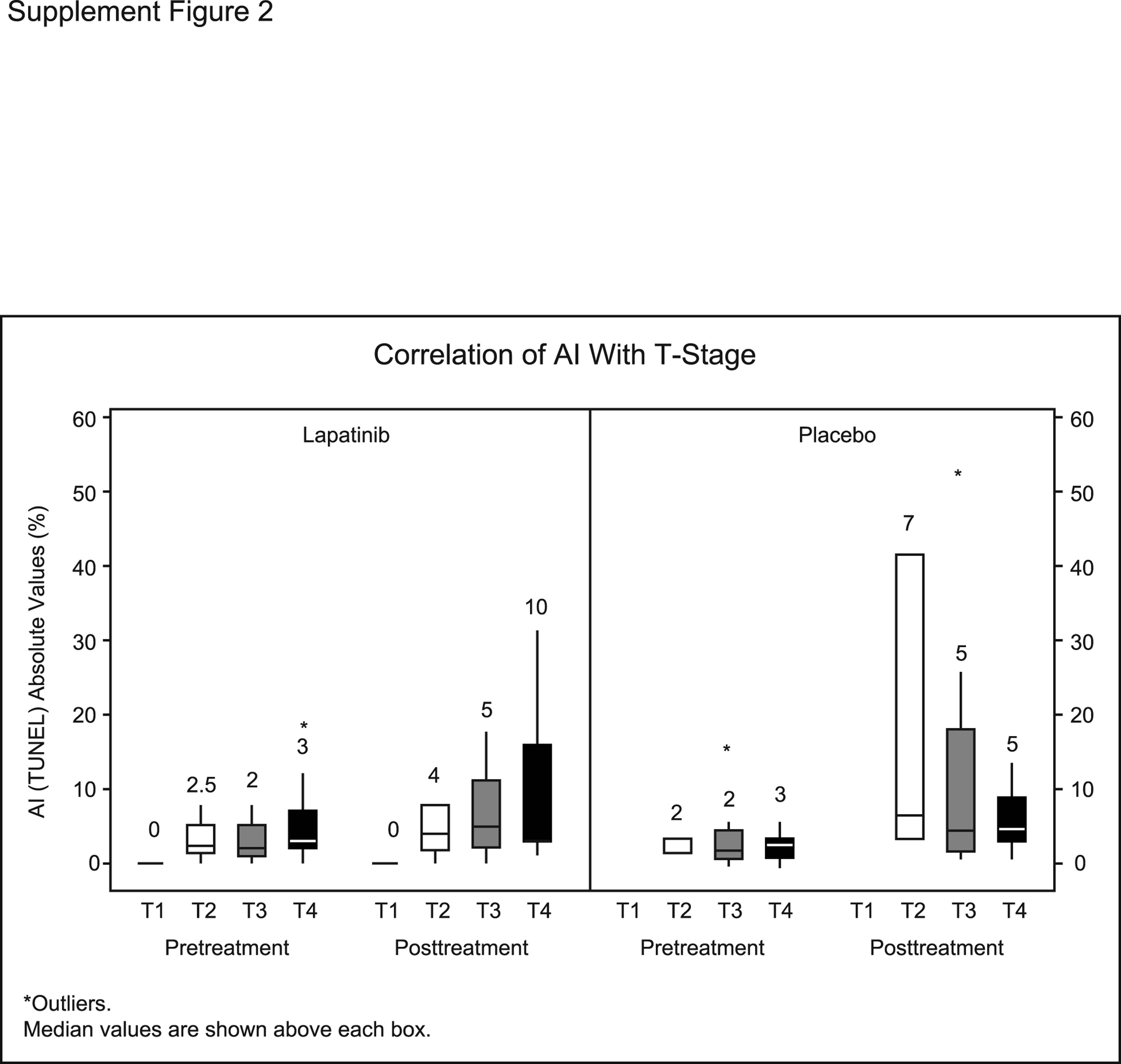

Supplement: Supplementary Figure 2 [file bjc2011237x2.tif]

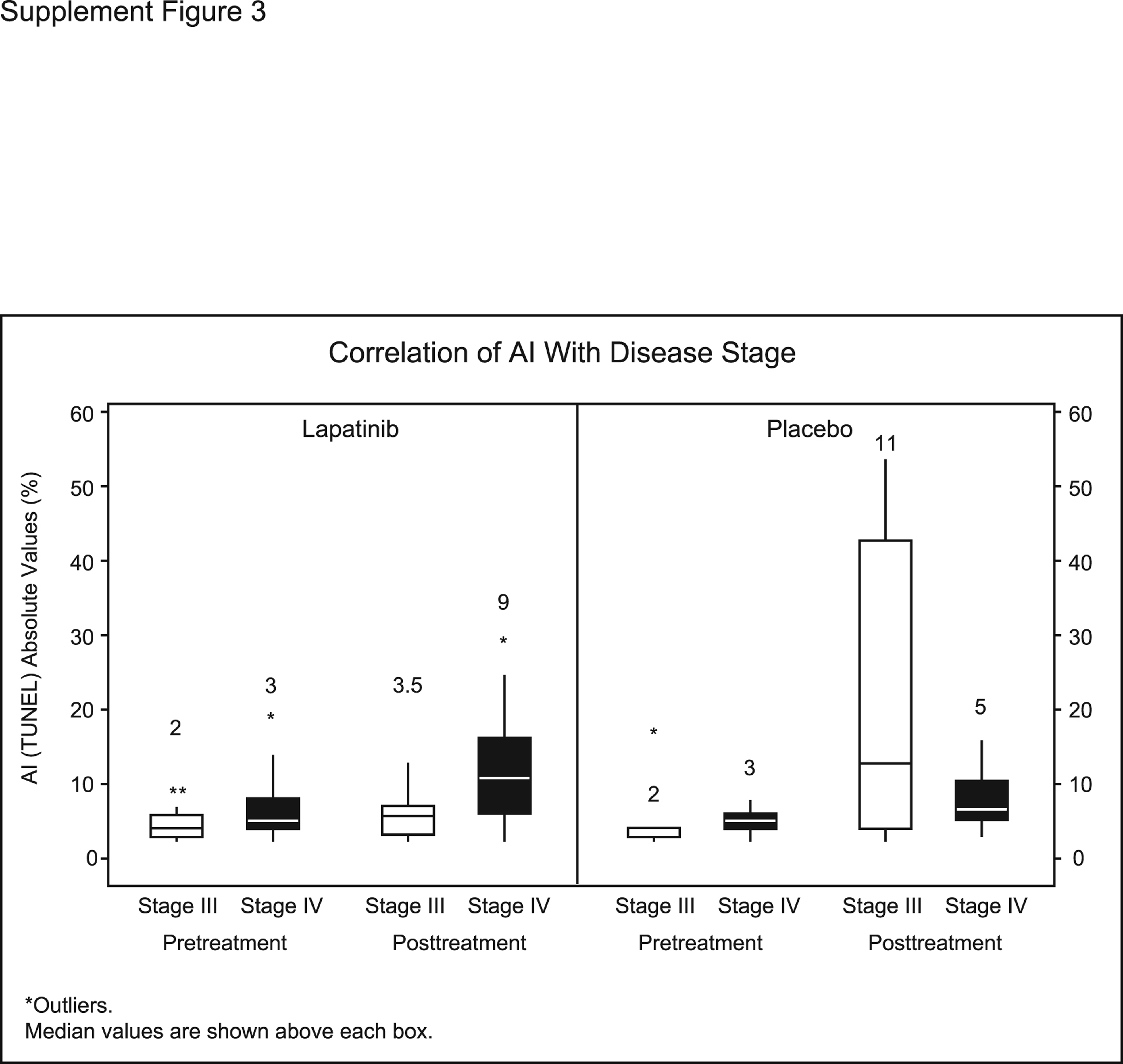

Supplement: Supplementary Figure 3 [file bjc2011237x3.tif]

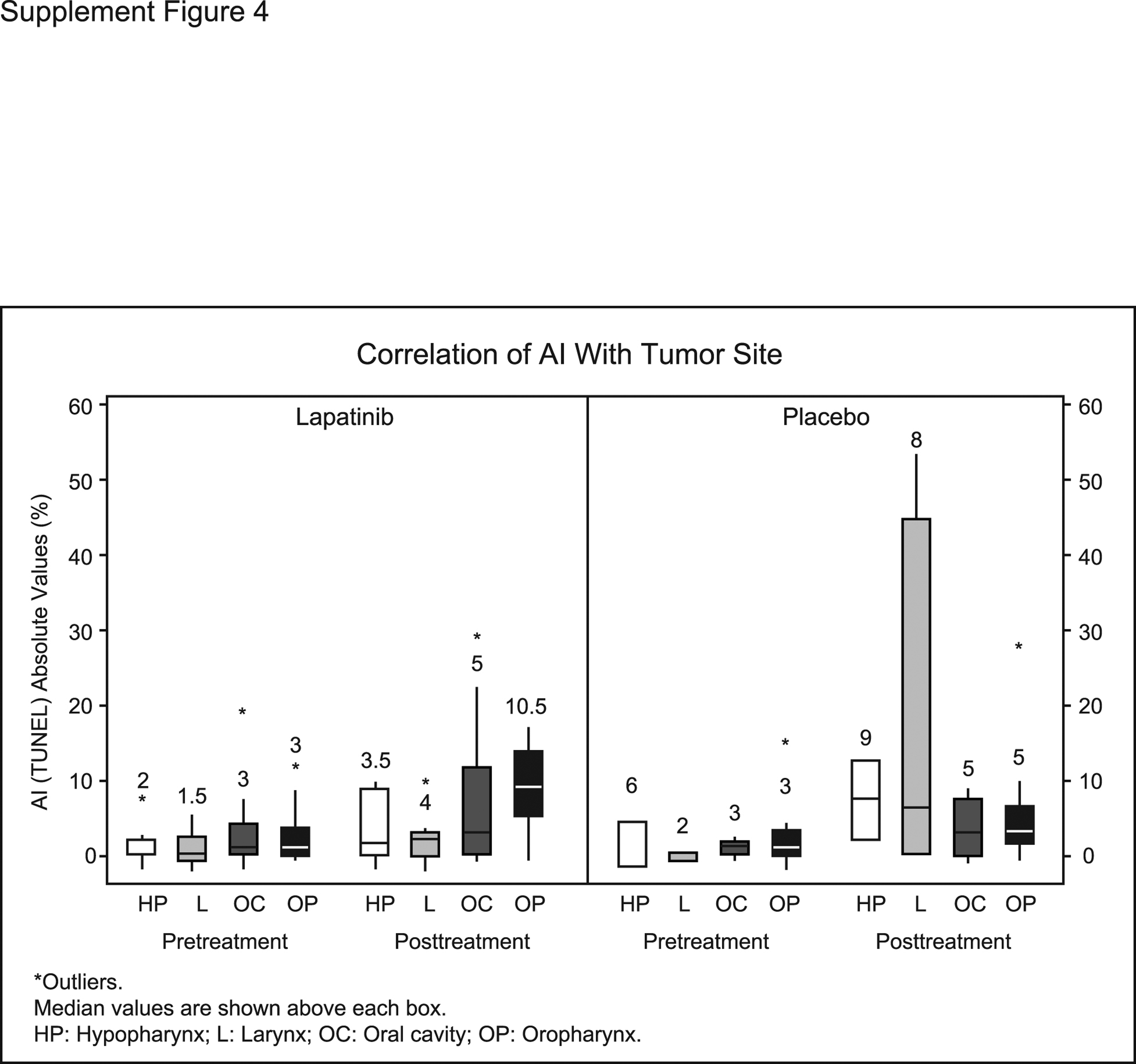

Supplement: Supplementary Figure 4 [file bjc2011237x4.tif]

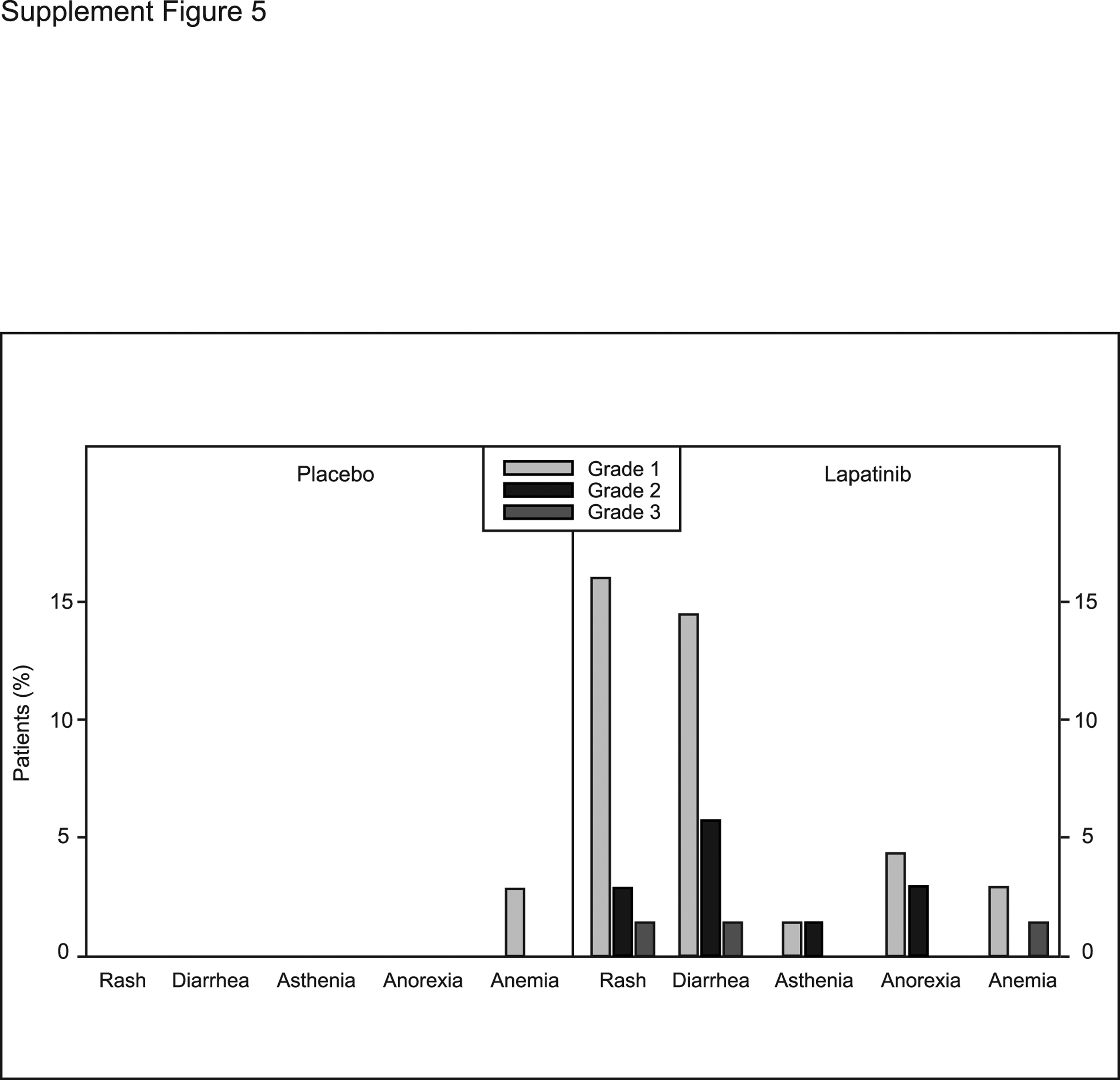

Supplement: Supplementary Figure 5 [file bjc2011237x5.tif]
