# Supplementary material for: Effects of lapatinib monotherapy: results of a randomised phase II study in therapy-naive patients with locally advanced squamous cell carcinoma of the head and neck
Source: Br J Cancer. 2011 Aug 9;105(5):618–27. doi: 10.1038/bjc.2011.237 (PMC3188940; doi:10.1038/bjc.2011.237)
Supplement: Supplementary Figure Legends [file bjc2011237x6.doc]

**SUPPLEMENTARY FIGURE LEGENDS**

**Supplementary Fig 1.** **Box and whisker plot of changes in AI pretreatment and posttreatment.** Abbreviation: TUNEL, terminal deoxynucleotidyl transferase-mediated deoxyuridine triphosphate nick-end labeling.

**Supplementary Fig 2.** **Correlation of AI is shown with T stage.** Abbreviation: TUNEL, terminal deoxynucleotidyl transferase-mediated deoxyuridine triphosphate nick-end labeling.

**Supplementary Fig 3.** **Correlation of AI is shown with Disease stage.** Abbreviation: TUNEL, terminal deoxynucleotidyl transferase-mediated deoxyuridine triphosphate nick-end labeling.

**Supplementary Fig 4.** **Correlation of AI is shown with Tumor site.** Abbreviation: TUNEL, terminal deoxynucleotidyl transferase-mediated deoxyuridine triphosphate nick-end labeling.

**Supplementary Fig 5.** Bar chart of adverse events (AEs) and associated toxicity grade reported during the monotherapy phase.
