# Supplementary material for: Effects of lapatinib monotherapy: results of a randomised phase II study in therapy-naive patients with locally advanced squamous cell carcinoma of the head and neck
Source: Br J Cancer. 2011 Aug 9;105(5):618–27. doi: 10.1038/bjc.2011.237 (PMC3188940; doi:10.1038/bjc.2011.237)
Supplement: Supplementary Table 1–4 [file bjc2011237x7.doc]

**Supplementary Table 1**. Details of antibodies used in IHC and FISH analysis

| **Assay** | **Anti-body** | **Clone** | **Specificity** | **dilution** | **-ve control** | **+ve control** | **Counter-stain** | **Detection system** |
| --- | --- | --- | --- | --- | --- | --- | --- | --- |
| TUNEL | N/A | Terminal Deoxy-nucleotidyl Transferase for DNA cleavage | DNA strand breaks by TdT labeling | N/A | Method negative | Hyperplastic lymph node | Methyl Green | ISH- Peroxidase |
| Cleaved Caspase 3 (Asp175) | Cell Signaling #9661 | N/A | Cleaved Caspase-3 (Asp175) Antibody detects endogenous levels of the large fragment (17/19 kDa) of activated caspase-3 resulting from cleavage adjacent to Asp175. | 1:25 | Method negative | Hyperplastic lymph node | Methyl Green | High Sensitivity Polymer |
| Ki-67 | Dako #M7240 | MIB-1 | epitope encoded by a 66bp repetitive element in the Ki-67 gene | 1:100 | Method negative | Tonsil or lymph node | Methyl Green | High Sensitivity Polymer |
| EGFR | Dako pharmDx kit | 2-18C9 | structural epitope in the extracellular cysteine-rich region of the molecule spanning sub-domain S2 and proximal to the transmembrane region | Pre-dilute | Cell pellet control in kit | Cell pellet control in kit | Methyl Green | High Sensitivity Polymer |
| p-EGFR | Cell Signaling #2234 | N/A | Tyr 1068 | 1:100 | Method negative | Breast Ca | Methyl Green | High Sensitivity Polymer |
| HER2 | Vector Laboratories #VP-C380 | CB11 | internal  domain of the C-erbB-2 oncoprotein. | 1:80 | Method negative | Breast Ca | Methyl Green | High Sensitivity Polymer |
| p-HER2 | Cell Signaling #2243 | 6B12 | Tyr1221/1222 | 1:200 | Method negative | Breast Ca | Methyl Green | High Sensitivity Polymer |
| P53 | Dako #M7001 | DO-7 | Amino acids 1 - 45 | 1:80 | Method negative | Breast Ca | Haematoxylin | High Sensitivity Polymer |
| Probe |  | Target Band Region | Target Chromosome |  |  |  | Counter-stain | Detection  System |
| LSI EGFR | 05J48-001 | 7p12 | 7 | N/A | N/A | Amplified Breast Cancer | DAPI | Spectrum Orange |
| LSI HER-2 | 02J01-035 | 17q11.2-q12 | 17 | N/A | N/A | Amplified Breast Cancer | DAPI | Spectrum Orange |

Key: IHC: Immunohistochemistry; TUNEL: terminal deoxynucleotidyl transferase–mediated deoxyuridine

triphosphate-biotin nick-end labeling; FISH: Fluorescent in situ hybridization

**Supplementary Table 2.** Basic clinical and pathological characteristics of all responders on lapatinib treatment

| **Baseline characteristics** | **P16** | **EGFR**  **IHC** | **HER2**  **IHC** | **EGFR**  **FISH** | **HER2 FISH** | **Pre Rx pEGFR** | **Post Rx pEGFR** | **Pre Rx**  **AI %** | **Post Rx**  **AI%** | **Pre Rx**  **PI%** | **Post Rx**  **PI%** | **Monotherapy**  **Response** | **CRT**  **Response** |
| --- | --- | --- | --- | --- | --- | --- | --- | --- | --- | --- | --- | --- | --- |
| Male, 49, T2N1,  Oral Cavity,  P16 -ve | -VE | 2+ | 0 | Non-  amplified | Non-  amplified | 2+ | 1+ | 0 | 5 | 27 | 25 | CR | CR |
| Male, 77, T3N0,  Oral cavity,  P16+ve | +VE | 3+ | 0 | Non-  amplified | Non-  amplified | 1+ | 1+ | 1 | 1 | 38 | 45 | PR | SD |
| Male, 58, T1N3, Hypopharynx, P16+ve | +VE | 3+ | 3+ | Amplified | Amplified | 0 | 2+ | 0 | 0 | 83 | 81 | PR | PD |
| Male, 65, T3N1, Glottic,  P16(nk) | Not Known | 3+ | 1+ | Amplified | Non-  amplified | 1+ | 1+ | 2 | 4 | 62 | 27 | PR | WD |

**Supplementary Table 3.** Summary of AEs Experienced by 15% or More Subjects in Either Treatment Group (Safety Population)

| **Preferred term** | **Number (%) of subjects** | |
| --- | --- | --- |
|  | **Placebo**  **N=36** | **Lapatinib**  **N=69** |
| **Any event** | 36 (100) | 69 (100) |
| Mucositis | 24 (67) | 48 (70) |
| Asthenia | 17 (47) | 23 (33) |
| Odynophagia | 13 (36) | 23 (33) |
| Dysphagia | 13 (36) | 22 (32) |
| Nausea | 9 (25) | 21 (30) |
| Rash | 5 (14) | 21 (30) |
| Vomiting | 14 (39) | 18 (26) |
| Diarrhea | 2 (6) | 18 (26) |
| Dry mouth | 8 (22) | 15 (22) |
| Anorexia | 11 (31) | 14 (20) |
| Neutropenia | 10 (28) | 13 (19) |
| Radiation skin injury | 8 (22) | 13 (19) |
| Constipation | 9 (25) | 12 (17) |
| Pharyngolaryngeal pain | 6 (17) | 12 (17) |
| Skin reaction | 7 (19) | 11 (16) |
| Dysgeusia | 4 (11) | 11 (16) |
| Dysphonia | 8 (22) | 8 (12) |
| Pain | 6 (17) | 8 (12) |
| Pyrexia | 6 (17) | 7 (10) |
| Leukopenia | 7 (19) | 4 (6) |

**Supplementary Table 4: Summary of SAEs Started During or After CRT Phase (Safety Population)**

| **Preferred Term** | **Number (%) of Subjects** | |
| --- | --- | --- |
|  | **Placebo**  **N=36** | **Lapatinib**  **N=69** |
| **Any event** | **13 (36)** | **13 (19)** |
| Mucosal inflammation | 2 (6) | 3 (4) |
| Constipation | 0 | 2 (3) |
| Respiratory tract infection | 2 (6) | 1 (1) |
| Asthenia | 0 | 1 (1) |
| Bipolar disorder | 0 | 1 (1) |
| Cardio-respiratory arrest | 0 | 1 (1) |
| Chronic obstructive pulmonary disease | 0 | 1 (1) |
| Dehydration | 0 | 1 (1) |
| General physical health deterioration | 0 | 1 (1) |
| Intestinal perforation | 0 | 1 (1) |
| Peripheral embolism | 0 | 1 (1) |
| Renal failure | 0 | 1 (1) |
| Skin ulcer | 0 | 1 (1) |
| Sudden death | 0 | 1 (1) |
| Pyrexia | 2 (6) | 0 |
| Vomiting | 2 (6) | 0 |
| Diabetic ketoacidosis | 2 (6) | 0 |
| Electrolyte imbalance | 1 (3) | 0 |
| Lobar pneumonia | 1 (3) | 0 |
| Neutropenia | 1 (3) | 0 |
| Parotitis | 1 (3) | 0 |
| Pneumonia aspiration | 1 (3) | 0 |
| Post procedural haemorrhage | 1 (3) | 0 |
| Renal impairment | 1 (3) | 0 |
| Sepsis | 1 (3) | 0 |
| Septic shock | 1 (3) | 0 |
| Upper respiratory tract infection | 1 (3) | 0 |
| Ventricular fibrillation | 1 (3) | 0 |
| Weight decreased | 1 (3) | 0 |
